# How Are Physical Activity Behaviors and Cardiovascular Risk Factors Associated with Characteristics of the Built and Social Residential Environment?

**DOI:** 10.1371/journal.pone.0126010

**Published:** 2015-06-02

**Authors:** Michael Eichinger, Sylvia Titze, Bernd Haditsch, Thomas E. Dorner, Willibald J. Stronegger

**Affiliations:** 1 Institute of Social Medicine and Epidemiology, Medical University of Graz, Universitätsstrasse 6, 8010, Graz, Austria; 2 Institute of Sport Science, University of Graz, Mozartgasse 14, 8010, Graz, Austria; 3 Styrian District Health Insurance Fund, Friedrichgasse 18, 8010, Graz, Austria; 4 Institute of Social Medicine, Medical University of Vienna, Rooseveltplatz 3, 1090, Vienna, Austria; 5 Institute of Social Medicine and Epidemiology, Medical University of Graz, Universitätsstrasse 6, 8010, Graz, Austria; University of Tolima, COLOMBIA

## Abstract

**Background:**

The aim of our study was to identify perceptions of built and social residential characteristics and their association with behaviors such as physical activity (PA), nutrition and smoking and with cardiovascular risk factors (elevated BMI and fasting blood glucose).

**Methods:**

Among participants of a preventive medical checkup at an Austrian District Health Insurance Fund (n=904, response rate = 82.2%, 42% women, 18-91 years) self-reported and measured data were collected.

**Results:**

Total PA was positively associated with the presence of trees along the streets and high levels of pro-physical activity social modeling (SM) and it was negatively related to perceived safety from crime. More leisure-time PA was associated with higher levels of cycling/walking infrastructure and high levels of SM. PA for transportation was positively related to high levels of connectivity and high levels of SM. Better behavioral cardiovascular risk factor profiles (smoking and nutrition) were associated with high levels of SM and high levels of total PA. Lower BMI values were associated with high levels of infrastructure and high levels of SM.

**Conclusions:**

Both built and social residential characteristics are important correlates of PA as well as of major cardiovascular risk factors besides PA.

## Background

It is widely recognized that non-communicable diseases—cardiovascular diseases (CVDs) being an important subgroup—constitute a large part of the global burden of disease [1]. In 2008 approximately 17.3 million people died from CVDs, representing 30% of all global deaths [2]. Due to demographic changes, the fraction of the burden of disease that is accounted for by coronary heart disease (CHD) will further increase in many countries. For example, the aging population will lead to a sizeable increase in CHD incidence, prevalence, mortality and costs associated with CHD mortality and morbidity [3,4,5,6].

Unhealthy behaviors, such as physical inactivity, unhealthy diet, smoking, and cardiovascular risk factors such as overweight and obesity, high serum cholesterol, elevated blood glucose and high blood pressure, have been identified to substantially contribute to CHD morbidity and mortality. A majority of these causes of CHD are lifestyle-related and hence modifiable [7,8,9,10,11,12]. Results of an Austrian study revealed that a large proportion of the spatial variance in CVD mortality can be explained by the spatial distribution of classical CVD risk factors and psycho-social risk factors [13].

It is widely recognized that in order to positively modify cardiovascular risk factors and risk behaviors, a multilevel approach is needed. Interventions should therefore target individuals, social environments, built environments and policies [14]. Some frameworks such as the social-ecological model, and theories such as the social cognitive theory, already propose a wide range of measures of how to sustainably promote healthy behavior with a focus on individual and social approaches. Based on the above-mentioned models, the scientific community shifted its focus beyond the psycho-social level and included environmental contexts as correlates of cardiovascular risk factors and risk behaviors—including low levels of physical activity (PA) as one important component [15,16,17,18].

Associations between built residential characteristics and different dimensions of PA have been recently documented. Street connectivity and access to sidewalks have been shown to be positively associated with PA [19,20,17,21]. The general attractiveness of the residential environment, the presence of enjoyable scenery, the degree of greenness and the perceived levels of safety in the neighborhood have been shown to be important correlates of PA [22,23,21,24]. Empirical work has moreover demonstrated the importance of social modeling and social support for high levels of PA [23,21,25].

Recent studies suggest that built residential characteristics are moreover associated with cardiovascular risk factors/risk behaviors other than low levels of PA. Residents of highly walkable neighborhoods and neighborhoods characterized by high connectivity levels had lower BMIs than their counterparts in worse neighborhoods [17,26]. A study by Mujahid et al. [27] has demonstrated that subjects living in highly walkable neighborhoods with higher levels of safety and social cohesion had a lower probability of being hypertensive. While these associations were robust when adjusting for socioeconomic indicators, they were attenuated after adjustment for race/ethnicity.

Both theoretical considerations and empirical studies have had the aim to determine the extent to which PA mediates the influence of the quality of the residential environment on health outcomes; however, results remain inconsistent in the literature. A strand of literature suggests that both PA and exposure to a pleasant environment independently contribute to health outcomes [28,29], while empirical results about the association between residential environment and health outcomes in other studies suggest a strong mediating effect of PA [24].

The aim of this study was to identify associations between perceived characteristics of the built and social residential environment and cardiovascular risk behaviors/risk factors. While the present study does not encompass a complete risk behavior/risk factor profile, we did succeed in capturing many of the most important cardiovascular risk behaviors (low levels of PA, nutritional and smoking behavior) and risk factors (BMI and fasting blood glucose).

## Methods

### Study design

A cross-sectional survey was conducted among participants of a preventive medical checkup at the Styrian District Health Insurance Fund (*Steiermärkische Gebietskrankenkasse*, Austria) between March and May 2012. Self-reported data were obtained by means of a questionnaire that was filled in by the participants during the checkup. In the questionnaire we asked about characteristics of the built and social residential environment, about different types of PA as well as different dimensions of health and health behavior. In addition, the medical staff recorded the participants' fasting blood sugar levels.

### Ethics statement

The study protocol, the survey as well as the consent procedure were approved by the ethics committee of the Medical University of Graz prior to the start of the study (No. 24–026 ex 11/12). Upon arrival at the facilities of the District Health Insurance Fund all participants of the preventive medical checkup were informed about the study in detail. At this stage verbal informed consent was obtained from all individuals that were willing to participate in the study. After both the participants and the medical staff had completed the survey, participants had the opportunity to anonymously cast the survey into a box. The cast of the survey into the box can be interpreted as the participant’s agreement to participate in the study. All information that was entered into the survey by the participants and the medical staff was anonymous. The identification of individuals through the data that had been provided was not possible at any time.

### Independent variables

Characteristics of the built residential environment were assessed using the European environmental questionnaire ALPHA-Long (ALPHA-L) in German, which includes items on distances to local facilities, walking/cycling infrastructure in the neighborhood and its maintenance, neighborhood safety, walking/cycling networks, home environment and the workplace/study environment. The different items were assessed by the use of rating scales. For details of the development of the questionnaire, see Spittaels et al. [15]. The questionnaire has good reliability and predictive validity for PA [16,30].

Characteristics of the social environment were assessed on five-point Likert scales ranging from 1 (strongly disagree) to 5 (strongly agree). Six items adopted from Wood et al. [31] were included:

- Living in my neighborhood gives me a sense of community.
- I regularly stop and talk with people in my neighborhood.
- It is easy to make friends in my neighborhood.
- I regularly seek advice from people in my neighborhood.
- I regularly borrow things and exchange favors with my neighbors.
- I would be willing to work together with others on something to improve the living environment in my neighborhood.

Social support and social modeling with regard to PA were assessed on a five-point rating scale ranging from 1 (strongly disagree) to 5 (strongly agree) by the following statements: “Many individuals in my family/among my friends encourage me to be physically active.”/”Many individuals in my family/among my friends are often physically active.” In a previous study [32] we tested the test-retest reliability of the item “social support from friends” and found an acceptable agreement (0.730). The items social modeling (“number of adults in home and close friends who exercise regularly”)—typically the questions are separated for family members and for friends—are frequently used in studies investigating determinants of physical activity and showed acceptable test-retest reliability [33]. The same is true for family support.

The individual socio-economic status (SES) was assessed by a cumulative score which included the equivalence income of the respondent and the educational status, and was divided into quintiles ranging from 1 (lowest) to 5 (highest). Equivalence incomes were calculated using the reported net income of the household while adjusting for the household size. Educational status was assessed using the following seven categories (sample percentages in parentheses): no compulsory school (0.2%), compulsory school (8.8%), apprentice training (43.5%), intermediate vocational degree (14.0%), high school diploma (20.0%), university of applied sciences/university with first degree (11.7%), university with doctorate (1.7%). Age was divided into three categories: <35 (22.0%), 35–55 (48.9%), >55 (29.1%) years. Detailed descriptive statistics of the sample are presented in Table 1.

**Table 1:** Sample characteristics.

		N	%
Gender	Male	523	57.85
	Female	381	42.15
Age (years)	<35	198	21.90
	35–55	445	49.23
	>55	261	28.87
Individual-level SES^a^ (quintiles)	1st (lowest)	113	12.50
	2nd	175	19.36
	3rd	183	20.24
	4th	131	14.49
	5th (highest)	302	33.41
Education	no compulsory school	2	0.22
	compulsory school	77	8.52
	apprentice training	397	43.92
	intermediate vocational degree	125	13.83
	high school diploma	180	19.91
	university of applied sciences/ university with first degree	107	11.84
	university with doctorate	16	1.77
Total sample		904	

Note: Percentages do not always add up to 100% due to rounding errors.

^a^ Socio-economic status.

In order to control for the degree of urbanity of the neighborhood, we dichotomized the urban-rural gradient. We used the neighborhoods' postal codes provided by the participants to create a variable with the following categories: urban/living in Graz (capital of the province of Styria, population of about 300,000) (1) and rural/living outside of Graz (2).

### Dependent variables

Levels of PA were assessed using the long form of the International Physical Activity Questionnaire (IPAQ-L) in German. For the analyses the domains (i) work-related PA, (ii) PA related to transportation and (iii) leisure-time PA were included. The domain household- and yard-related PA was excluded because the objective was to find correlates of non-domestic PA. The literature suggests that the IPAQ-L questionnaire has both reliability and validity characteristics that are comparable to other established self-reported measures of PA [34]. The PA reported in sections (i)–(iii) was used to calculate continuous scores in MET (metabolic equivalent of task)-minutes/week, as suggested in the literature [35]. Additionally, a total PA score was derived by adding up the scores of the three sections.

The body mass index (BMI) of all participants was calculated using self-reported measures of body weight and body height. Self-reported smoking behavior was assessed using the following categories (sample percentages in parentheses): smoking occasionally (7.4%), smoking <10 (7.5%), 11–20 (11.2%), >20 (2.6%) cigarettes/day, ceased smoking (29.3%), never smoked (42.2%). Nutritional behavior was identified by questions about the participants' consumption frequencies (“How many days per week do you normally eat...?”) of the following items: (i) meat, (ii) vegetables, (iii) fruit and (iv) convenience food, with answers ranging from 0 to 7 days. The smoking and nutritional behavior were used to calculate a cumulative score (with low scores corresponding to better cardiovascular risk factor profiles) capturing these two important behavioral dimensions of the participants' cardiovascular risk factor profiles.

In order to determine fasting blood glucose levels (mg/dL) the medical staff performed venous blood sampling of all participants on the day of the routine check-up. All blood samples were analyzed at the laboratory facilities of the Styrian District Health Insurance Fund.

### Data analysis

A specific feature of the present study was the two-step approach in our analyses. We identified relevant dimensions of the residential environment in a first step and analyzed potential associations between the dimensions of the environment and our dependent variables in a second step. All the analyses were adjusted for sex, age and the individual-level SES.

All analyses were performed using Stata/SE 11.2 software for Mac(R). In order to identify fundamental aspects of the perceived residential environment, a comprehensive list of items concerning built and social characteristics of the residential environment were subjected to four separate factor analyses using principal component analysis and applying orthogonal rotation (Varimax). Only factors with eigenvalues equal to or higher than 1 were retained. In order to assess the internal consistency of the extracted factors Cronbach's alpha was calculated for the items included in the factors. Cumulative scores encompassing the items of each factor were calculated and normalized to a scale ranging from 1 to 10. In the subsequent regression analyses, the extracted factors were used as independent variables.

Before performing the regression analyses, the bivariate relationships between the extracted factors and PA as well as the aforementioned dimensions of health were analyzed. The associations between the built and social residential characteristics and both PA behavior and health dimensions were analyzed using stepwise multiple linear regression models. A significance level of 0.1 was applied for the removal of predictors from the model. In order to discover potential mediating effects of the PA behavior, all base models of the health-related regressions were additionally adjusted for total PA levels in a second step (only the final models which include total PA as a control are reported). If appropriate, dependent variables were logarithmized to achieve an approximate Gaussian distribution.

## Results

### Descriptive statistics

Out of a total of 1100 participants of the preventive medical checkup that were asked to participate in the study, 904 returned the questionnaire (response rate = 82.2%). The final sample showed the following composition with respect to gender (male: 523 (57.9%), female: 381 (42.2%)) and educational status (with high school diploma: 303 (33.5%), without: 601 (66.5%)). The final sample had a mean age of 47.1 years (SD: 14.1), a mean BMI of 25.2 kg/m^2^ (SD: 4.1) and a mean fasting blood glucose of 98.7 mg/dL (SD: 17.3). Table 1 shows descriptive statistics for the final sample.

### Factor analysis of the residential environment variables

For the characteristics of the built environment we derived six factors explaining 65.1% (factor 1) and 64.0% (factors 2–6) of the variance respectively.

1. *Perceived distance to local facilities* (Cronbach alpha = 0.90, eigenvalue (EV) = 4.56, 65.1% of variance explained): “distance to local shops”, “distance to supermarket”, “distance to local services, e.g. post office, library”, “distance to restaurant/pub”, “distance to fast-food restaurant”, “distance to bus stop/train station”, “distance to leisure/sport facilities”.

2. *Perceived availability/maintenance of cycling/walking infrastructure* (Cronbach alpha = 0.86, EV = 4.04, 21.3% of variance explained): “availability of sidewalks in neighborhood”, “availability of pedestrian zones/trails in neighborhood”, “availability of bike lanes in neighborhood”, “availability of cycle routes separated from traffic in neighborhood”, “maintenance of sidewalks in neighborhood”, “maintenance of cycle paths in neighborhood”, “maintenance of playgrounds/parks in neighborhood”.

3. *Perceived connectivity* (Cronbach alpha = 0.66, EV = 2.07, 10.9% of variance explained): “cycling is quicker than driving in neighborhood”, “density of road junctions in neighborhood”, “different routes for walking/cycling from place to place in neighborhood”.

4. *Perceived safety with regards to traffic* (Cronbach alpha = 0.79, EV = 2.25, 11.8% of variance explained): “possibility to cross busy streets safely”, “insecurity while walking due to traffic”, “insecurity while cycling due to traffic”.

5. *Perceived safety from crime* (Cronbach alpha = 0.74, EV = 2.36, 12.4% of variance explained): “danger during the day because of crime”, “danger during the night because of crime”, “litter or graffiti in the streets”, “presence of badly maintained/unoccupied buildings”.

6. *Overall residential quality* (Cronbach alpha = 0.43, EV = 1.44, 7.6% of variance explained): “neighborhood as pleasant environment for walking/cycling”, “presence of trees along the streets”.

As the Cronbach alpha was <0.60 for factor 6, we used the two individual items instead of the factor for all subsequent linear regression models.

For the characteristics of the social environment we derived three factors explaining 66% (factor 7) and 78.7% (factors 8–9) of the variance respectively.

7. *Social cohesion* (Cronbach alpha = 0.86, EV = 3.30, 66% of variance explained): A complete list of the items (i)-(v) that were included in this factor is included in section 2.2 of this paper.

8. *Pro-physical activity social support* (Cronbach alpha = 0.77, EV = 1.69, 42.2% of variance explained): “many individuals in my family/of my friends strongly suggest to me to be physically active”.

9. *Pro-physical activity social modeling* (Cronbach alpha = 0.60, EV = 1.45, 36.4% of variance explained): “many individuals in my family/of my friends are often physically active”.

### Linear regression analysis of physical activity behaviors

Total PA was positively associated with the presence of trees along the streets in the neighborhood and high levels of pro-physical activity social modeling (Table 2) and was negatively related to perceived safety from crime. Leisure-time PA was positively associated with levels of perceived availability/maintenance of cycling/walking infrastructure and levels of pro-physical activity social modeling (Table 2). Negative relationships were found between leisure-time PA and perceived connectivity, perceived safety with regards to traffic and perceived safety from crime. PA for transportation was positively related to high levels of perceived connectivity and high levels of pro-physical activity social modeling. A negative association was found between transportation-related PA and pro-physical activity social support. No associations were found between work-related PA and the included built and social characteristics of the residential environment.

**Table 2:** Cardiovascular risk factors/behaviors by built and social residential characteristics as well as individual characteristics—final stepwise multiple linear regression model results (1)^a^.

	PA—total [MET-min/week, log]	PA—leisure [MET-min/week, log]	PA—transport [MET-min/week, log]	PA—work [MET-min/week, log]
	Coef.	Coef.	Coef.	Coef.
Perceived distance to local facilities				
Perceived availability/maintenance of cycling/walking infrastructure		0.087**		
Perceived connectivity		-0.071**	0.083**	
Perceived safety with regards to traffic		-0.061*		
Perceived safety from crime	-0.136**	-0.089*		
Neighborhood as pleasant environment for walking/cycling				
Presence of trees along the streets	0.132**			
Social cohesion				
Pro-physical activity social support			-0.048*	
Pro-physical activity social modeling	0.090**	0.131**	0.061*	
R^2^	0.11	0.11	0.08	0.14
Sample n	720	635	567	360

^a^ Only statistically significant coefficients that remained in the final model of the stepwise multiple linear regression are displayed. Analysis adjusted for gender, age and individual-level socio-economic status.

* p < 0,05;

** p < 0,01

No relationship between the different dimensions of PA and both the distance to local facilities, the item “neighborhood as pleasant environment for walking/cycling” and social cohesion was found.

The results of the stepwise multiple linear regression models for men and women separately are presented in Tables 3 and 4.

**Table 3:** Cardiovascular risk factors/behaviors by built and social residential characteristics as well as individual characteristics—final stepwise multiple linear regression model results—Women^a^.

	PA—total [MET-min/week, log]	PA—leisure [MET-min/week, log]	PA—transport [MET-min/week, log]	PA—work [MET-min/week, log]	CV risk factor index [log]	BMI [kg/m^2^, log]	FBG [mg/dL, log]
	Coef.	Coef.	Coef.	Coef.	Coef.	Coef.	Coef.
Perceived distance to local facilities			-0.093**		-0.017*		0.008**
Perceived availability/maintenance of cycling/walking infrastructure		0.099**				-0.017**	
Perceived connectivity		-0.107**					
Perceived safety with regards to traffic							
Perceived safety from crime	-0.143**	-0.124**					
Neighborhood as pleasant environment for walking/cycling							
Presence of trees along the streets							
Social cohesion						0.009*	
Pro-physical activity social support			-0.065*			0.009*	
Pro-physical activity social modeling	0.120**	0.104**	0.089*		-0.035**		
PA—total (MET-min/week, log)							
R^2^	0.09	0.13	0.11	0.13	0.20	0.24	0.16
Sample n	291	263	226	134	285	263	276

^a^ Only statistically significant coefficients that remained in the final model of the stepwise multiple linear regression are displayed FBG fasting blood glucose; analysis adjusted for age and individual-level socio-economic status;

* p < 0,05;

** p < 0,01.

**Table 4:** Cardiovascular risk factors/behaviors by built and social residential characteristics as well as individual characteristics—final stepwise multiple linear regression model results—Men^a^.

	PA—total [MET-min/week, log]	PA—leisure [MET-min/week, log]	PA—transport [MET-min/week, log]	PA—work [MET-min/week, log]	CV risk factor index [log]	BMI [kg/m^2^, log]	FBG [mg/dL, log]
	Coef.	Coef.	Coef.	Coef.	Coef.	Coef.	Coef.
Perceived distance to local facilities				0.120*			
Perceived availability/maintenance of cycling/walking infrastructure		0.058*					
Perceived connectivity			0.095**				
Perceived safety with regards to traffic		-0.088**					
Perceived safety from crime	-0.102*						
Neighborhood as pleasant environment for walking/cycling							
Presence of trees along the streets	0.150*						
Social cohesion	-0.010**			-0.163**			
Pro-physical activity social support						0.010**	0.008*
Pro-physical activity social modeling	0.083*	0.135**			-0.022**	-0.012**	
PA—total (MET-min/week, log)							
R^2^	0.15	0.10	0.09	0.20	0.14	0.11	0.11
Sample n	429	372	341	226	421	373	384

^a^ Only statistically significant coefficients that remained in the final model of the stepwise multiple linear regression are displayed FBG fasting blood glucose; analysis adjusted for age and individual-level socio-economic status;

* p < 0,05;

** p < 0,01.

### Linear regression analysis of other cardiovascular risk behaviors and risk factors

Better behavioral cardiovascular risk factor profiles (with lower values on the *CV risk factor index* corresponding to a better behavioral cardiovascular risk factor profile) were associated with high levels of social modeling and high levels of total PA (Table 5). Lower BMI values were associated with high levels of perceived satisfaction with the availability/maintenance of cycling/walking infrastructure, low levels of social cohesion, low levels of pro-physical activity social support and high levels of social modeling. Low fasting blood glucose was related to low levels of perceived distance to local facilities and low levels of pro-physical activity social support.

**Table 5:** Cardiovascular risk factors/behaviors by built and social residential characteristics as well as individual characteristics—final stepwise multiple linear regression model results (2)^a^.

	CV risk factor index [log]	BMI [kg/m^2^, log]	FBG [mg/dL, log]
	Coef.	Coef.	Coef.
Perceived distance to local facilities			0.006*
Perceived availability/maintenance of cycling/walking infrastructure		-0.007*	
Perceived connectivity			
Perceived safety with regards to traffic			
Perceived safety from crime			
Neighborhood as pleasant environment for walking/cycling			
Presence of trees along the streets			
Social cohesion		0.006*	
Pro-physical activity social support		0.010**	0.005*
Pro-physical activity social modeling	-0.029**	-0.012**	
PA—total (MET-min/week, log)	-0.016*		
R^2^	0.21	0.22	0.22
Sample n	706	636	660

^a^ Only statistically significant coefficients that remained in the final model of the stepwise multiple linear regression are displayed FBG fasting blood glucose; analysis adjusted for gender, age and individual-level socio-economic status;

* p < 0,05;

** p < 0,01.

No relationship was found between these cardiovascular risk behaviors/risk factors and perceived connectivity, perceived safety with regards to traffic, perceived safety from crime, neighborhood as pleasant environment for walking/cycling and presence of trees along streets.

## Discussion

### Perception of the built and social environment

We were able to explain the perception of the residential environment by eight independent factors—five pertaining to characteristics of the built environment and three concerning social dimensions. While some of the derived factors pertaining to characteristics of the built environment are similar to the scores used in Bucksch et al. [30], who also used the ALPHA environmental questionnaire, for some factors there are notable differences. The “Distance to local facilities” factor combines the distance to the various local facilities that people use on a day-to-day basis. This factor only differs from the “Distance to local facilities” score in Bucksch et al. [30] insofar as their score encompasses an item on open recreation areas in addition to our items. The “Perceived availability and maintenance of cycling/walking infrastructure” factor concerns the availability and maintenance of infrastructure that facilitates walking and cycling within the quarter, including the presence of bike lanes, sidewalks, pedestrian zones, etc. While Bucksch et al. [30] use two separate scores, the results of the factor analysis of the present sample supported the inclusion of both availability and maintenance measures in one factor describing the general functionality of a neighborhood with regards to walking and cycling, which is jointly determined by the availability and the maintenance of adequate infrastructure. The third factor was termed “Perceived connectivity”, which combines factors like the density of road junctions or the availability of different routes from one place to another that facilitate walking and cycling in the neighborhood. While Bucksch et al. [30] additionally included the “Many shortcuts for walking” item in their connectivity score, the results of the present factor analysis justified the inclusion of the “Cycling is quicker than driving during the day” item. The “Perceived safety with regards to traffic” factor covers perceived insecurity during walking and cycling specifically due to traffic of high intensity. The fifth identified dimension concerns both perceived insecurity due to crime, poor maintenance of buildings within the neighborhood and the presence of litter and graffiti. This factor differs from the scores used in Bucksch et al. [30] as it combines both items from their “Safety from crime” sum score and their measure for “Aesthetics” into one factor capturing the degree to which an area is safe and attractive. The first factor concerning social dimensions combines a variety of items concerning social relations with neighbors and was named “Social cohesion”. The “Pro-physical activity social support” dimension summarizes the presence of people that verbally suggest higher levels of PA. The last factor concerns the presence of role models that are physically active themselves and was named “Pro-physical activity social modeling”.

### Physical activity and the residential environment

The measures of PA that we assessed showed a great variety of associations with dimensions of the residential environment. A complete overview of the associations found is presented in Table 2.

As disparities in cardiovascular risk behaviors/risk factors across quarters might well be due to individual-level characteristics (i.e. neighborhood composition) rather than neighborhood characteristics [36], we controlled for various individual-level indicators. As the associations persisted after including potential confounders (individual-level SES, age, sex) in the model, disparities in risk behaviors/risk factors may not be simply attributed to higher socio-economic levels in wealthier quarters.

Both total PA and leisure-time PA were found to be negatively related to perceived safety. Similar findings are reported in Titze [22]. A possible explanation for the unexpected direction of this association in our study is the fact that individuals that exercise more outside also have a greater opportunity to observe events that lower the perceived level of safety within the neighborhood. More generally, this may mean that not only perceived safety influences patterns of PA but that PA also influences the individual's perception of the quarter's safety [22,23]. An analog explanation seems reasonable for our finding that leisure time PA was negatively associated with perceived safety with regards to traffic within the quarter.

Higher levels of total PA were strongly associated with the presence of trees along the streets in the neighborhood. While we were not able to show statistically significant associations for the three sub-dimensions of PA—specifically not for leisure-time PA-, our findings involving total PA are generally in line with results presented in Giles-Corti et al. [21], who found that individuals were more likely to walk for recreational purposes if they perceived the quarter as attractive and interesting. King et al. [23] report that the presence of enjoyable scenery was associated with more PA in women. Sugiyama et al. [24] report a correlation between the greenness—our measure of trees along the street being one element of the overall greenness of the quarter—and walking for recreation.

High levels of total PA as well as the sub-dimensions for both transportation-related PA and leisure-time PA were associated with high levels of pro-physical activity social modeling. These results are consistent with other studies that report that frequently observing others exercising in the quarter came along with more PA in women [23], that knowing people that walk themselves was associated with a higher likelihood to walk for recreation oneself [21] and that higher levels of active transportation were related to more social modeling in the Belgian subsample [25].

We found that leisure-time PA was positively associated with the availability and maintenance of cycling/walking infrastructure. This is in line with Giles-Corti et al. [21] who found—although not statistically significant (95% CI: 0.99–2.03, p-value: 0.058)—that walking for recreation—one dimension of leisure-time PA—is more likely if there was access to sidewalks in the neighborhood.

Leisure-time PA was negatively related to high levels of perceived connectivity. The available literature [17,20] and intuition would suggest—to the contrary—a positive relationship. This paradox effect in our study might be due to a confounding effect of the degree of urbanity of the neighborhood, i.e. the degree of urbanity of the neighborhood might have an independent effect on the level of leisure-time PA. This hypothesis is connected to findings in recent studies that suggest that the fact of whether a person lives in a rural or an urban area might have an important effect on PA levels. For example Moore et al. [37] documented that moderate-to-vigorous PA levels for rural youth were significantly lower than for urban youth. Our sample consisted of participants from both urban and rural counties. While we controlled for individual-level SES and focused on characteristics of the immediate residential environment, we did not include any measure to account for different levels of urbanity of a residential neighborhood in our base model. Upon inclusion of a control variable for the degree of the neighborhoods' urbanity the negative relationship between the perceived connectivity and leisure-time PA remained (the coefficient declined somewhat from -0.071 (p-value < 0.01) to -0.079 (p-value < 0.01)). It should be noted that the measure of urbanity derived from the postal codes provided by participants is a rather crude proxy and does not reflect the full urban-rural gradient. While our result does not support the proposed confounding effect of the degree of urbanity of the neighborhood, the potential existence of such a confounder warrants future research on more sophisticated measures of urbanity and on a possible independent effect of the degree of urbanity of the neighborhood on PA.

We found that transportation-related PA was positively associated with high levels of perceived connectivity. Similar results have been reported in the literature: Quarters that are characterized by high street connectivity were associated with a higher prevalence of walking, meeting PA recommendations [19] as well as higher levels of PA for transportation [20].

### Other cardiovascular risk behaviors and risk factors and the residential environment

The cardiovascular risk behaviors/risk factors that we assessed were associated with various dimensions of the residential environment. The observed associations persisted once we controlled for the level of overall PA (in addition to the demographic and socioeconomic variables). While total PA was associated with better behavioral cardiovascular risk factor profiles, it does not seem to be the only mediatory variable between different characteristics of the residential environment and cardiovascular risk factors, as the inclusion of total PA into the model did not substantially alter the other associations. Our findings are consistent with results reported in a closely connected strand of literature [28,29] suggesting that both PA *and* residential characteristics, e.g. the exposure to pleasant environment, independently contribute to health outcomes; however the overall results remain inconsistent in the literature. Empirical results about the association between residential environment and health outcomes in some studies [24] suggest a strong mediating effect of PA.

Better cardiovascular risk factor profiles were related to high levels of pro-physical activity social modeling. This association persisted even after including total PA as a control variable. This might be due to (i) the existence of a direct link between pro-physical activity social modeling and the determinants of the cardiovascular risk factor profile or (ii) the presence of a third variable that mediates the association. Future research is needed to further elucidate possible causal pathways.

Better cardiovascular risk factor profiles were associated with higher levels of total PA. This is consistent with related literature documenting influences of PA levels on cardiovascular risk factor profiles. Oja et al. [11] provide a review of evidence documenting positive associations between cycling for different purposes and cardiovascular fitness and cardiovascular risk factors.

Lower BMI levels were strongly associated with high-level satisfaction with the perceived availability and maintenance of cycling/walking infrastructure. Consistent with our findings, Saelens et al. [17] report that residents of highly walkable neighborhoods—the availability of sidewalks and bike trails being one dimension of walkability—had lower BMIs than residents of low-walkability residential areas.

Moreover, we found that the BMI level was negatively associated with pro-physical activity social modeling. This is consistent with our result showing that high levels of pro-physical activity social modeling were associated with better outcomes for both various dimensions of PA and better cardiovascular risk factor profiles.

### Strengths and limitations of the study

Our study has a number of strengths and limitations worth noting, concerning both the study design and the measurements. Firstly, concerning measurements, it is important to note that we were able to employ detailed instruments for surveying characteristics of the built environment (ALPHA-L) and PA behavior (IPAQ-L). Therefore, discriminating between different components of PA in an internationally recognized and standardized manner as well as distinguishing different dimensions underlying the built environment was possible. Other complex, multidimensional concepts (e.g. nutritional behavior, mental health) were assessed in a less detailed manner.

Secondly, the inclusion of self-reported measures might be regarded as a drawback due to the subjective nature of the responses. We would like to take a more differentiated stand on this. In our opinion, subjective and objective measures constitute complementarities rather than substitutes and have to be used together to grasp the full complexity of the world surrounding us. Hence, future research should concentrate both (i) on identifying new subjective and objective measures for characteristics of the residential environment and (ii) on investigating innovative ways of integrating measures of both types in order to get detailed pictures of the residential environment.

Our sample was drawn from participants of a voluntary preventive medical check-up. In total, around 40% of Austria's population take part in such preventive medical check-ups in three years. As known from previous analyses, subjects aged over 40 years, with higher education, higher income, without migration background, and those with a chronic disease have a higher chance of participating in health check-ups [38]. Due to the probable existence of selection bias, the possibility to generalize from our analyses to the general Austrian population is limited. The fact that our sample is limited to participants from the city of Graz and surrounding counties additionally limits the generalizability of our findings. This is of special importance due to the fact that there is a substantial spatial gradient in both cardiovascular risk factors and disease prevalences in Austria [13]. Großschädl et al. [39] report findings on the presence of a clear east-west gradient for the prevalence of obesity with higher rates in Eastern Austria. Due to the fact that we obtained a relatively high response rate, we could ensure that our results are representative for participants of preventive medical check-ups in Styria.

Furthermore, it should be noted that the survey was cross-sectional. Therefore, we were only able to report correlations. The investigation of underlying causal relationships has to be confirmed using additional information and longitudinal study designs.

## Conclusions

While there is increasing evidence for the effect of the built residential characteristics on the PA behavior of individuals, the influence of the social environment and the simultaneous evaluation of multiple risk behaviors/risk factors has received less attention to date. The present study attempted to fill this gap by deliberately including social characteristics in the study design and investigating smoking and nutritional behavior—two central behavioral dimensions of the participants' cardiovascular risk factor profiles-, BMI and fasting blood glucose as well as PA behavior. While the present study does not encompass a complete cardiovascular risk factor profile, we captured many of the most important risk factors and found most of them to be associated with characteristics of the built and social residential environment.

While there exists a growing amount of studies investigating possible links between both built and social residential characteristics and PA behavior, the number of articles on the influence of neighborhood characteristics on other cardiovascular risk factors, e.g. BMI or fasting blood glucose, remains small. Future studies should embrace a greater variety of cardiovascular risk behaviors/risk factors in order to elucidate the complete array of (causal) pathways between residential characteristics and risk factors.

Moreover, future research should focus on integrating both innovative objective and subjective measures for characteristics of the residential environment. As a majority of the existing evidence documenting associations between characteristics of the residential environment and risk factors as well as risk behaviors is mainly cross-sectional [40], future research should use prospective longitudinal designs or natural experiments in order to be able to acquire knowledge about causal links rather than merely about observed associations. With the ultimate goal of our research in mind—solving real-world problems—future research should last but not least strive to draw conclusions in such a way that they can more easily serve as guidelines for political decision makers.
